# Optimization of Nanoparticles for Smart Drug Delivery: A Review

**DOI:** 10.3390/nano11112790

**Published:** 2021-10-21

**Authors:** Lina Jia, Peng Zhang, Hongyan Sun, Yuguo Dai, Shuzhang Liang, Xue Bai, Lin Feng

**Affiliations:** 1 School of Mechanical Engineering and Automation, Beihang University, Beijing 100191, China; Linajia@163.com (L.J.); zp989dream@163.com (P.Z.); hongyansun@buaa.edu.cn (H.S.); daiyuguo5612@g.ecc.u-tokyo.ac.jp (Y.D.); liangsz13nq@buaa.edu.cn (S.L.); 2 Beijing Advanced Innovation Center for Biomedical Engineering, Beihang University, Beijing 100191, China

**Keywords:** biocompatibility, targeting efficiency, drug loading rate

## Abstract

Nanoparticle delivery systems have good application prospects in the treatment of various diseases, especially in cancer treatment. The effect of drug delivery is regulated by the properties of nanoparticles. There have been many studies focusing on optimizing the structure of nanoparticles in recent years, and a series of achievements have been made. This review summarizes the optimization strategies of nanoparticles from three aspects—improving biocompatibility, increasing the targeting efficiency of nanoparticles, and improving the drug loading rate of nanoparticles—aiming to provide some theoretical reference for the subsequent drug delivery of nanoparticles.

## 1. Introduction

**Figure 1:**
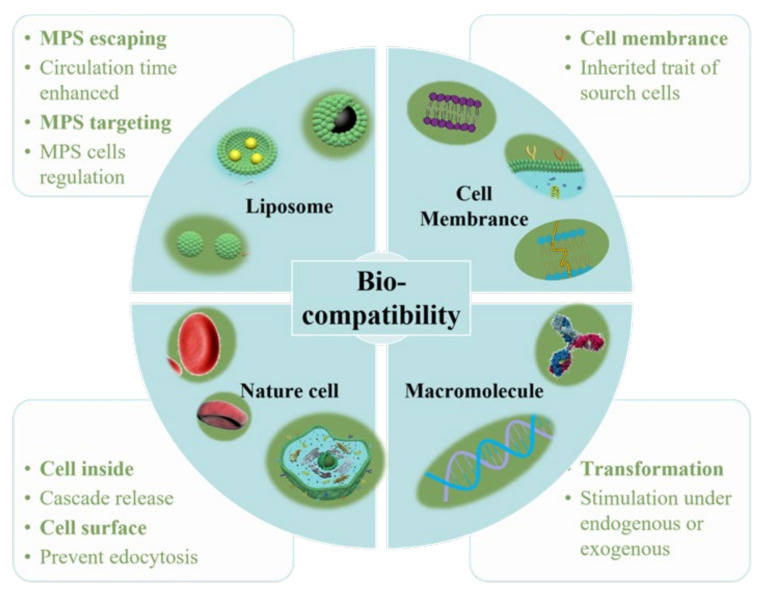
Methods for increasing the biocompatibility of micro-nano robots.

As a “functional molecular device” that can be freely controlled in a liquid environment, nanoparticles have gradually developed into a new generation of multi-functional micro-nano control tools. In particular, it has great application prospects in biomedical fields, such as targeted drug delivery and release, disease diagnosis and so on. Multi-functional nanoparticles work as an important branch of medical micro-nano robots. For the diffusion of nanoparticles, however, micro-nano robots have better initiative, as they control their targeted movement and drug unloading through endogenous or exogenous stimulation [1]. Nanoparticles have excellent performance in in vitro experiments, but they will encounter a complex biological environment at the risk of being eliminated once entering the physiological environment in the body. Firstly, as a “foreign body”, nanoparticles will be quickly eliminated by the immune system. Secondly, unlike cells, nanoparticles cannot actively sense and target the disease environment, thus limiting the accumulation of nanoparticles in the lesions. Cell membrane–camouflaged nanoparticles comprise a type of synthetic nanoparticle as the core, wrapped with a layer of natural cell membranes, such as red blood cell membranes, immune cell membranes, cancer cell membranes and platelet membranes. Different types of cell membranes will give nanoparticles different biological behaviors [2]. Thus, when constructing the nanoparticle delivery system, biocompatibility must be taken into account. Indeed, the researchers have modified the surface of conventional nanomaterials and optimized their composition in a variety of ways to improve their biocompatibility (Figure 1).

**Figure 2:**
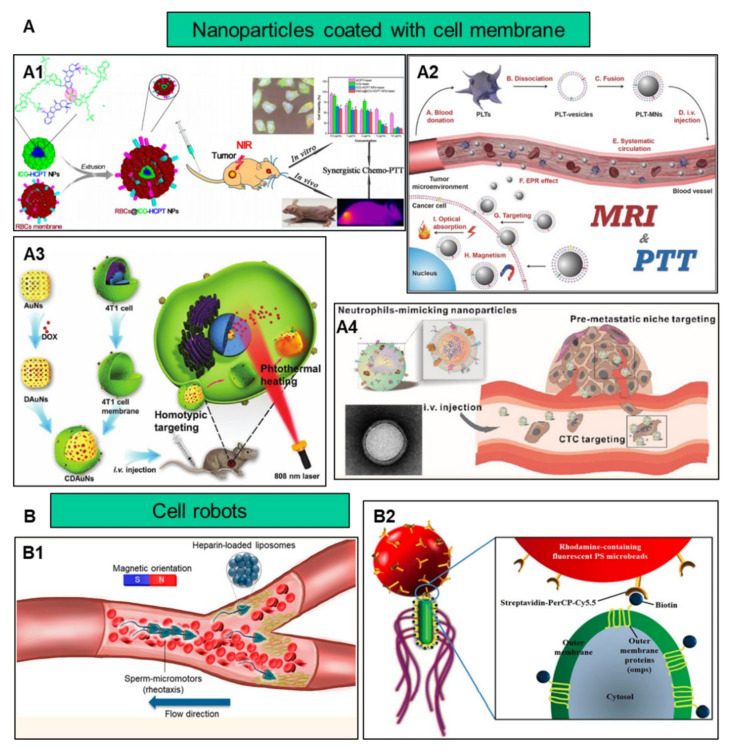
Illustrations of nanoparticles coated with cell membrane and cell robot for improving the targeting efficiency. (**A**) Nanoparticles coated with cell membrane. (**A1**) Light/pH-triggered biomimetic red blood cell membrane–camouflaged small molecular drug assemblies for imaging-guided combinational chemo-photothermal therapy. Adapted with permission [7]. Copyright ACS Appl Mater, 2019. (**A2**) Antitumor platelet-mimicking magnetic nanoparticles. Adapted with permission [8]. Copyright Advanced Functional Materials, 2017. (**A3**) 4T1 cancer cell membrane–coated gold nanocage for the hyperthermia-triggered release of DOX and homotypic targeted therapy of breast tumor growth and metastasis. Adapted with permission [9]. Copyright Advanced Functional Materials, 2017. (**A4**) Nanoparticles coated with neutrophil membranes can effectively treat cancer metastasis. Adapted with permission [10]. Copyright ACS Nano, 2017. (**B**) Cell robots. (**B1**) Sperm micromotors for cargo delivery through flowing blood. Adapted with permission [11]. Copyright ACS Nano, 2020. (**B2**) New paradigm for tumor theranostic methodology, using bacteria-based microrobots. Adapted with permission [12]. Copyright Sci Rep, 2013.

In addition to improving biocompatibility, increasing the target efficiency of nanoparticles will help to further increase nanoparticle efficacy by promoting preferential accumulation at the site of interest. Utilizing the characteristics of different cells, such as chemotaxis of macrophages and the homing effect of cancer cells, nanoparticles and cell characteristics are combined to increase the targeting function of nanoparticles (Figure 2).

Nanoparticles as drug carriers have important research and application value in medicine. The level of drug loading determines the frequency of administration, which is of outstanding significance in reducing clinical costs and side effects and improving the quality of life of patients [3]. According to the existing nano-drug delivery system, the nano-drug carrier system can be divided into four categories as per the drug loading mechanism: molecular-level loading system, loading on the surface of nanoparticles, matrix loading system and cavity loading system [4]. Carriers can be divided into organic carriers and inorganic carriers, which have attracted widespread interest, due to their superior characteristics and promising applications in the field of biomedicine [5]. Nanoscale drug-delivery systems based on organic and inorganic carriers provide an infinite matrix of nanoparticles with different properties. This allows carrier nano-drug delivery systems to perform more complex functions in physiological systems [6]. However, the low drug-carrying capacity of carrier drugs (usually <10 wt%) limits the accumulation of effective drugs. It also promotes the development of carrier-free nano-drug delivery systems (usually greater than 80 wt%).

This review focuses on summarizing the recent advances in the field of optimization of nanoparticles for smart drug delivery, including improving biocompatibility, increasing the targeting efficiency and improving the drug loading rate. We hope to provide information for researchers to construct an excellent nanovector for clinical applications.

## 2. Improving Biocompatibility

### 2.1. Liposomal Formulations

Liposomes are artificial membranes with bilayer structures similar to cell membranes. When amphoteric molecules, such as phospholipids and spingolipids, are dispersed in the aqueous phase, their hydrophobic tails tend to cluster together to avoid the aqueous phase, while the hydrophilic heads are exposed to it, forming closed vesicles with bilayer structures [13]. Liposomes can encapsulate not only lipophilic substances, but also water-soluble substances and amphoteric substances. In addition, liposomes are naturally biodegradable, non-toxic and non-immunogenic, making them highly biocompatible [14]. Both of those make liposomes suitable vectors for a wide range of therapeutic applications. Liposomes have been approved by the U.S. Food and Drug Administration (FDA) for clinical use, such as doxorubicin hydrochloride liposome [15], siRNA drug (Onpattro) [16] and mRNA [17]. As a carrier, liposomes in the organism begin their fate with the reaction of proteins in the serum, such as opsonin [18] or high-density lipoprotein [19]. Then, they are recognized and ingested by the mononuclear phagocyte system (MPS) [20], and finally degrade in the cell to release the cargo [21]. Most of the time, however, liposomes need to be avoided by MPS in order to target the lesion more effectively [22]. Therefore, we will discuss the properties and functions of liposomes from two aspects: MPS targeting and MPS escaping. For the MPS targeting strategy, liposomes can be used for immunotherapy, tissue regeneration, anti-tumor and other macrophage related diseases. For the MPS escaping strategy, liposomes can be used for directly targeting diseased cells. Liposome targeting or escaping of monocytes and macrophages can be achieved by modifying the composition of liposomes. 

MPS can present antigens, regulate inflammation, and secrete cytokines to regulate tissue regeneration. Therefore, targeting MPS is an important strategy for directly targeting diseased cells, aimed at increasing the circulation time of liposomes in vivo and avoiding the rapid clearance by MPS. Liposomes’ composition can be optimized by inserting ligands, such as peptides, antibodies, and other biomacromolecules, for disease detection, imaging or treatment (Table 1).

### 2.2. Cubosomes

**Figure 3:**
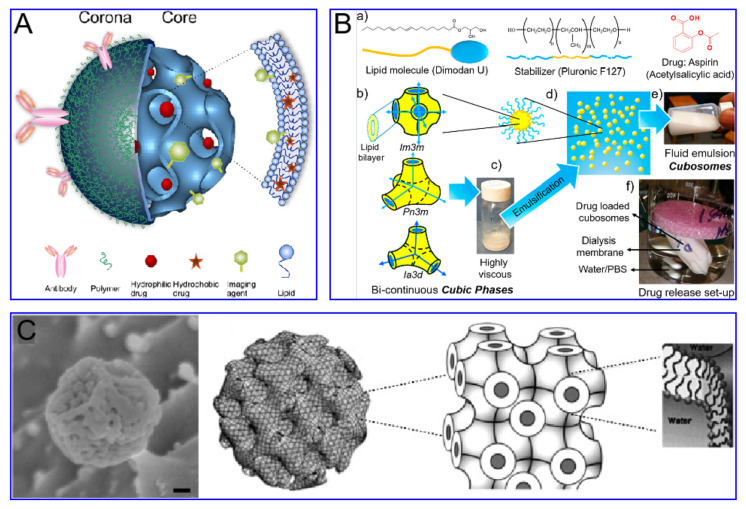
Cubosomes for therapeutic agent delivery; (**A**) Typical cubosome schematic. Reprinted with permission [13]. Copyright (2019) American Chemical Society. (**B**) Cubosomes were used to transport small molecule drugs to cancer tissue. Reprinted with permission [14]. Copyright (2013) Adv Drug Deliv Rev. (**C**) Cubosomes were used to delivery biological macromolecular drugs for immunotherapy. Reprinted with permission [15]. Copyright (2015) American Chemical Society.

Cubosomes are a lipid bilayer self-assembled by amphiphilic lipid molecules, which further forms a stereoscopic structure with zero mean surface curvature by twisting, cycling and arranging in space according to a cubic lattice. They have been extensively studied in drug delivery (Figure 3). Compared to liposomes, the hydrophobic volume of cubosomes is larger. For the particle size of 100 nm, the volume fraction of the cubosome is 0.59 nm^3^ and that of the liposome is 0.18 nm^3^. This feature endows the cube with higher drug loading efficiency, especially for poorly water-soluble drugs. Moreover, cubosomes have a rather high viscosity (composite viscosity ranges from 10^4^ to 10^5^ Pa·s) to a resistance to rupture, ensuring that they are more robust and stable. The cubosome can efficiently load adriamycin and only releases them in an acidic microenvironment, where tumor cells are present, to kill tumor cells. This method not only increases the killing effect of adriamycin on tumor cells, but also reduces the toxic and side effects of adriamycin on normal cells. Cubosomes have been reported to not only promote the proliferation of CD^8+^ and CD^4+^ T cells, but also induce the secretion of interferon-γ and Ova-specific antibodies. Therefore, cubes can also be used as an effective slow-release delivery system for vaccines. In addition, cubosomes also were used to transport small molecule drugs (both hydrophobic and hydrophilic), peptides, proteins, nucleic acids and imaging agents into cells through oral, intravenous, transdermal and mucosal administration. 

### 2.3. Cell Membrane Interface

Nanomaterials coated with cell membranes are regarded as a promising method of biomimetic particle engineering [47]. Natural cell derivatives, such as extracellular vesicles and membranes, can inherit many of the properties of their source cells. Therefore, by coating nanoparticles with these derivatives, the nanoparticles not only have natural biocompatibility, but also have functions similar to those of their source cells [48]. So, this top-down engineering approach can be applied to the development of new therapeutic strategies [49]. Membranes that can be used to coat nanomaterials have been reported to involve the red blood cells [50], immune cells [51], platelets [52], stem cells [53], macrophages [54] and cancer cells [55]. These membrane-interfacing nanomaterials have been reported for use in targeted therapy, vaccination, virus detection and many other fields (Table 2).

### 2.4. Nature Cell

Using the body’s own cells as a vehicle is advantageous because autologous cells are not only incomparably biocompatible, but they also have a built-in mechanism to move between tissues and navigate long distances around the body [88]. In order to avoid damage to the carrier cells by the loaded drugs, the drug loading rate of the cells must be limited [3]. To solve this problem, researchers wrap drugs in nanoparticles and deliver them to the lesion site either by attaching them to the cell surface or by endocytosis into the cell. Then, the loaded drugs can be released in response to either endogenous or exogenous stimuli [89]. Cells that have been reported for use as nanomaterial carriers include stem cells [90], leukocytes [91], red blood cells [92], and T cells [93]. Methods of endogenous stimulation include pH, reactive oxygen species (ROS), enzyme, hypoxia and so on [94]. The exogenous stimuli include near infrared radiation (NIR), magnetic field, ultrasound, etc. [95]. When used as drug carriers, erythrocytes have high encapsulation rates and long-term sustained release [96]. White blood cells (including monocytes, neutrophils, and lymphocytes) can actively cross biological barriers, such as tissue endothelium and the blood–brain barrier; they also have a tendency of chemoattraction toward the diseased site to inhibit infection, inflammation and tumor growth [97]. Because of this unique feature, they have the potential to carry drugs to other parts of the body that are inaccessible or difficult to reach through traditional drug delivery strategies.

### 2.5. Biomacromolecule

Micro-nano robots assembled with biomacromolecules for disease diagnosis and treatment refer to those robots that can deform and perform different functions under the conditions of endogenous or exogenous stimulation [89]. An endogenous stimulus condition refers to when a micro-nano robot enters an organism at the target position (usually the affected place), it is stimulated by the micro-environment of the target position that is different from normal tissues, and then deforms and performs the next function [98]. For example, precursors are now common in nervous system drugs, antitumor system drugs, and antiviral drugs [98]. These precursor drugs, which are not effective in vitro, can be activated by the target micro-environment after entering the human body to play a therapeutic role [99]. An exogenous stimulus condition refers to the type of robot that is deformed after receiving external stimuli [95]. For example, under ultraviolet light irradiation, gold nanoparticles with a small particle size and photocrosslinking agents on the surface can be polymerized into gold nanoparticles with a large particle size to increase photothermal conversion efficiency for the photothermal therapy of tumors [100]. Depending on their composition, these deformable biocompatible materials include synthetic polymers, proteins or DNA. Environmentally sensitive hydrogels refer to a class of polymer gels that can sense the slight changes or stimuli of the external environment (such as temperature, pH, light, electricity, pressure, etc.), and produce corresponding changes or even mutations in physical structures and chemical properties [101]. Because it can be degraded by organisms, it shows great application potential in the field of biomedicine. Environmentally sensitive hydrogels can be divided into temperature-sensitive hydrogels [102], pH-sensitive hydrogels [103], electrically responsive hydrogels [104], photosensitive hydrogels [105], magnetic-sensitive hydrogels [106], etc., according to the different responses to the external environment. Most deformable protein molecules in biomedical applications are deformable peptides, which can be divided into environmentally sensitive deformable peptides and cell membrane-penetrating peptides [107]. Drug delivery systems based on deformable peptides have been widely studied in tumor targeted therapy, for example, proton-driven tumor vaccine composed of deformable peptides for tumor immunotherapy [2], and an intracellular delivery system of chimeric peptides based on transmembrane peptides for acute liver injury in mice [3]. With the help of software, researchers can take tiny strands of DNA and fold them into complex structures, complete with components such as rotors and hinges that can move and perform tasks, such as drug delivery and cargo handling [108]. A nano-robot based on DNA origami technology can precisely locate tumor tissue and effectively inhibit tumor growth and metastasis. This nano-robot is made of a flat rectangular DNA origami board loaded with four thrombin molecules. Then, the DNA origami are rolled into hollow tubes that wrap the thrombin molecules inside and are locked by AS1411 aptamers. AS1411 aptamers can bind nuclides, which are highly expressed on vascular endothelial cells, and release the thrombin molecules, inducing local coagulation reaction in the tumor, and ultimately realize tumor coagulation necrosis and the treatment of the tumor [4]. In addition, DNA is also used to assemble a remotely controlled nanomechanical arm and transport a gold nanoparticle. The DNA-based robot also can be used to separate the cargo and transport them to the target zone, or even walk on the cell membrane to drive the cell motility.

## 3. Increasing the Targeting Efficiency

### 3.1. Cell Membrane

#### 3.1.1. Red Blood Cell Membrane

Tumors have unique enhanced permeability and retention (EPR), allowing them to preferentially capture nanoparticles and macromolecules. In order to accumulate a large quantity of nanoparticles at the tumor site, it is necessary to extend the circulation time of nanoparticles in the blood. Red blood cells (RBC) are the most abundant cell component [109]; they can survive for 120 days in the human body. Moreover, mature RBC lack a cell nucleus and some organelles, which are convenient for extraction and purification [110]. CD47, responsible for immune evasion, is expressed on the surface of RBC membranes. It can selectively bind to the SIRPα protein on macrophages, thereby avoiding being cleared by macrophages, prolonging the circulation time in the body, and increasing the accumulation at the target site [111]. 

The RBC membrane-coated nanoparticles can incorporate the unique advantages of natural RBC. Based on these characteristics, many research groups began to study the application of RBC membranes in cancer treatment. Zhang’s research team camouflaged the RBC membrane as nanoparticles for the first time, successfully prolonging the circulation time of nanoparticles [59]. Due to the EPR effect of the tumor, the accumulation of the RBC membrane-coated nanoparticles in the subcutaneous tumor site was significantly increased. In addition, some ligands that specifically bind to inflammatory tissues or tumor cells were inserted into the RBC membrane to increase the accumulation in targeted sites and reduce the uptake of immune cells. Common targeting ligands include peptides and small molecules, such as angiopep-2, folic acid [112], triphenylphosphonium, SHp (CLEVSRKNC), CDX (FKESWREARGTRIERG) and RGD [7]. Ye et al. developed a new type of erythrocyte membrane bionic combined therapy system by encapsulating 10-hydroxycamptothecin (10-HCPT) and indocyanine green (ICG) in the RBC membranes. Utilizing the camouflage function of RBC membranes, the particles significantly enhanced the accumulation at the tumor site through passive targeting and endocytosis [113].

Therefore, the RBC membrane cloaking strategy represents a powerful approach to nanomedicine for in vivo biomedical applications.

#### 3.1.2. Platelet Membrane

Platelets are the smallest circulating blood cells, fragments produced by mature megakaryocytes. Platelets play an important role in hemostasis, wound healing, inflammation and thrombosis after vascular injuries [114]. The antigens of platelet surface proteins can be instrumental in tumor metastasis by assembling to the surface of nanoparticles [115]. The platelet membrane-coated iron oxide nanoparticles reduced the uptake of macrophages, but greatly increased the uptake of nanoparticles by MCF-7 in vitro. The enhancement of immune evasion and the combination with cancer cells in the body system translated to the prolonged circulation time of nanoparticles and improved tumor targeting. Another study showed that platelet membrane-coated nanoparticles conduced to tumor targeting, which may be due to the interaction between platelet membranes and CD44 on breast cancer cell membranes [8]. In addition, platelet membrane-coated nanoparticles also have applications in other disease models. For example, Song et al. successfully demonstrated that it could be used for treatment of atherosclerosis by targeted drug delivery platforms [116]. Su et al. used the natural infarct homing ability of the platelet membrane to successfully achieve the targeted delivery of nanoparticles encapsulated by the platelet membrane to the heart after ischemic injuries for treatment [117].

#### 3.1.3. Cancer Cell Membrane

Compared with other blood cells, cancer cells boast many characteristics, such as unlimited replication, immune escape, and homologous targeting [118]. Cancer cells do not need to be obtained from the plasma of patients or donors in that they can be easily obtained from the in vitro culture [119]. 

The occurrence and metastasis of malignant tumors are usually caused by the immune escape of tumor cells. Cancer cells develop complex mechanisms to counteract or evade immune surveillance; for example, the overexpression of CD47 on the surface of breast cancer cell membranes may prevent them from being eliminated by the immune system. Nanoparticles encapsulated by cancer cell membranes are endowed with homotypic targeting properties. The study demonstrated that, compared with the uncoated tumor cell line MDA-MB-435 membrane, there was a significant accumulation of cancer cell membrane-coated nanoparticles in cancer cells [120]. Liu et al. constructed mesoporous silica nanoparticles with CaCO_3_ and cancer cell membranes, and the results showed that the cancer cell membrane coating contributed to the stability of the particles and the ability to accumulate at the tumor site [121]. Another group used lung cancer cell H460 membranes to bind two peptides, PD-L1 inhibitory peptide (TPP1) and MMP2 substrate peptide (PLGLLG), to coat superparamagnetic iron oxide nanoparticles through the homotypic effect of tumor cell membranes and the specific digestion of tumor-specific enzyme MMP2. The TPP1 peptide was delivered and released into the tumor micro-environment, showing a promising tumor treatment platform [122]. Compared with bare particles, nanoparticles wrapped in MCF-7 cell membranes significantly boost the absorption of nanoparticles by homologous cells [123]. In addition to targeting primary tumors, the use of homotypic targeting strategies can also deliver nanoparticles to metastatic tumors. The surface of 4T1 breast cancer cell membranes contains proteins and adhesion molecules related to metastasis and homotypic binding. These characteristics enable the cancer cell membrane-coated nanoparticles to effectively target metastatic breast cancer [9].

#### 3.1.4. Immune Cell Membrane

Immune cells can specifically target tumors. For example, the specific recognition protein on the membrane surface of activated T cells can recognize molecules on the tumor surface, thus exhibiting a high tumor affinity [124]. The immune recognition properties of T cells make their cell membranes a promising carrier for targeted drug delivery. However, due to the heterogeneity of tumors, the dual-targeting strategy holds prospects for tumor treatment [125].

Neutrophils are a type of white blood cells that can migrate through blood. Activated neutrophils tend to damage the inflammation sites spontaneously and exert their anti-inflammatory effects. By using the chemotactic behavior of neutrophils, it can play a huge part in the drug delivery system. There is evidence showing that neutrophils have the characteristics of circulating tumor cells [126], which possess micro-environment targeting properties through inherent cell adhesion molecules. Neutrophil membrane–coated polylactic acid glycolic acid (PLGA) nanoparticles can effectively capture circulating tumor cells and inhibit already-formed metastatic lesions [10].

Macrophages are the white blood cell population that accounts for the largest proportion of cancer tissues. Nanoparticles camouflaged by macrophage membranes can penetrate blood vessel barriers and recognize molecules on tumor cells. Tasciotti’s research group constructed a porous silica particle coated with a macrophage membrane for the first time. Functional molecules, such as CD45 and CD11a, are retained on the cell membrane, which helps preclude the uptake by macrophages, phagocytes or venous endothelial cells [127]. Wang et al. characterized the mechanism of macrophage membrane-coated nanoparticles targeting tumors. Blocking the receptors LFA-1 or CXCR1 and CXCR2 on the membrane will significantly restrain the recruitment of nanoparticles by inflamed tissues. In other words, the inflammation-related receptors on the membrane exert a pivotal role in the tumor homing effect [128]. These studies showed that nanoparticles camouflaged by macrophage membranes produce outstanding tumor targeting effects. However, the mechanism of tumor homing is controversial because cell adhesion, morphology, and cell–cell interaction are necessary for drug delivery, and nanoparticles camouflaged by macrophage membranes, not being living cells, cannot maintain all the complex biological functions of macrophages.

Compared with nanoparticles camouflaged by RBC membranes, those decorated with white blood cell membranes can not only prolong circulation in the body, but also actively target functional molecules on inflammation sites and cancer cell membranes. However, there are some issues that need to be considered. For example, as white blood cell membranes are mostly derived from immortalized cells, their biocompatibility is not as good as their RBC counterparts. In addition, the expression of specific histocompatibility complex molecules (MHC) on the leukocyte membrane may give rise to immunogenicity [129]. 

#### 3.1.5. Hybrid Membranes and Others

Integrating two or more kinds of cell membranes on the surface of nanoparticles can create unique biological properties of nanoparticles. For example, the RBC membrane and the cancer cell membrane are integrated to improve the efficiency of drug delivery. The platelet membrane and the cancer cell membrane are combined and modified with antibodies to increase the binding ability of cancer cells, which can reduce the interaction of homologous white blood cells and facilitate the separation of specific cancer cells. The gold nanowires are coated with the platelet membrane and the RBC, which can perform two different tasks at the same time: the platelet targets bacteria, while the RBC targets and neutralizes the toxins produced by the bacteria [130]. Another case showed that the cancer cell-RBC hybrid membrane-coated doxorubicin-loaded gold nanocage exhibits highly efficient accumulation in tumor sites due to the homologous targeting of the cancer cell membrane and decreased clearance due to the RBC membrane [131].

Bacterial membranes have a variety of immunogenic properties. Various pathogen-related molecular models can stimulate innate and adaptive immunity. Studies have reported that the presence of bacteria was detected in tumor tissues [132], and the coating of nanoparticles with bacterial membranes is capable of effectively targeting the tumor.

Although some progress has been made in nanoparticles camouflaged by cell membranes, some issues still exist. First of all, the source of cell membranes is very limited, and the separation and extraction steps are cumbersome, with a low yield. Secondly, the structure on the cell membrane is very complicated, and some components may induce an immune response. Finally, the control of cell membrane quality and safety also poses a problem. Nanoparticles for good cell membrane decoration require multidisciplinary cooperation.

### 3.2. Cell Robot

#### 3.2.1. Nanoparticles Coated with Bacteria

Bacteria have unique abilities that make them suitable as “small doctors”: they are self-driven (they can penetrate hard-to-reach tumor sites) and able to sense the local environment in response to external signals [133]. Bacteria use their cytochemical energy to drive flagella for fluid propulsion [134,135]. At a low Reynolds number, this kind of motion is unmatched by artificial propellers [135]. The speed and direction of movement of bacteria are affected by various external physical and chemical stimuli, and they actively migrate toward conditions beneficial to the [136], displaying navigability, phototaxis, chemotaxis and thermotaxis [137]. When designing drugs, the self-driving characteristics of the bacteria’s response to the environment can be utilized to guide them to a specific location inside the body.

A research group developed a bacterial robot, using the strong adsorption of bacteria to cy5.5-coated polystyrene microspheres. The experiments in vivo and in vitro confirmed that the robot was equipped with chemotaxis to tumor cell lysates or spheroids and tumor targeting [12]. Electromagnetically driven micro-nano robots are expected to be used for drug delivery [138,139]. The magnetic micro-nano robot can reach the designated position through the magnetic field generated by the coil so that it can be applied in the medical field. The magnetic micro-nano robot can move to the designated position of the large blood vessel, with a strong driving force because of the presence of an external magnetic field. Precise movement control requires the identification of blood vessel paths, but it is taxing to determine the path of small blood vessels, so precise cancer targeting is somewhat of an effort. Combining the characteristics of bacteria targeting their own tumors and electromagnetically driven micro-robots, Li et al. proposed a hybrid-driven micro-robot, which combined the macro-electromagnetic drive of large blood vessels and the micro-bacterial drive of small blood vessels to achieve macro-manipulation along the expected path. Through the chemotaxis of bacteria, the hybrid-driven micro-robot was microscopically manipulated toward the chemical attractant [140].

After that, there was a research project about using bacteria-driven biological hybrid micro swimmers for targeted drug delivery. Based on the affinity between bacterial type I fimbriae apex lectin molecules and mannose molecules on epithelial cells, they introduced a method for attaching bacteria to certain types of epithelial cells (mannose expressed on the membrane). Studies have shown that by expressing specific adhesion parts on the bacterial membrane, the bioadhesive movement system can be improved [141]. Saji Uthaman et al. directly used the specific interaction between streptavidin and biotin on bacteria to attach Salmonella flagellum to the surface of hyaluronic acid beads. After the bacteria were attached, their migration speed to tumor lysates rose drastically, which served as an eloquent testament to their potential in targeted anti-tumor therapy [142].

#### 3.2.2. Combination Nanoparticles with Cells

Compared with cells, the driving force generated by bacteria is small, making it difficult to accurately approach the target area [140]. In addition, bacteria are more toxic to the host, and some kinds of bacteria are not easy to culture in vitro. Immune cells, such as monocytes or macrophages can be used to carry drugs. They can penetrate the blood vessel barrier and exist as tumor-associated macrophages, occupying 70–80% of the tumor mass [143,144]. This feature qualifies them as vehicles to deliver therapeutic agents to tumors. There have been many studies based on macrophages or monocytes as carriers for drug delivery. For example, Choi used monocytes of gold nanoparticles to target tumors. Once close to the tumor, the cells were destroyed by near-infrared light, releasing nanoparticles to kill the tumor [144]. Chu’s group used macrophages to transport 7 nm gold nanorods to curb tumors [145]. Zhang’s group developed a macrophage delivery system carrying doxorubicin to treat breast cancer metastasis [59]. All these studies applied the tumor-homing characteristics of monocytes or macrophages to targeted drug delivery. However, the drug loading rate is low, and the targeting effect is not ideal. Dai et al. magnetized macrophages to reach designated locations under the external magnetic field and utilized the properties of macrophages to kill tumors [146,147]. Nguyen’s research group devised a new electromagnetic and macrophage-mediated drug delivery system. The anti-cancer drug paclitaxel (PTX) is wrapped in magnetic liposomes, which could be phagocytosed by mouse macrophages. The macrophages are recruited to tumors due to electromagnetic fields and the chemoattract properties of macrophages for dual targeting. This dual targeting system is expected to be developed into a potential cancer treatment strategy [148].

As the key cells of the immune response, neutrophils are crucial in eliminating the threat of infection through phagocytosis, degranulation, reactive oxygen species and neutrophils extracellular traps [149,150]. In the pathological process of inflammation, neutrophils can migrate across the blood–brain tumor barrier/blood–brain tumor barrier (BBB/BBTB) through chemotaxis along the inflammatory factor gradient [151,152]. Taking advantage of these characteristics, neutrophils were developed as drug carriers that target inflamed tumors [153,154,155]. However, this method has not completely cured cancer in mouse models. Zhang et al. reported a micro-robot constructed by the phagocytosis of natural neutrophils on the drug-loaded magnetic nanogel wrapped in *E. coli* membranes. The camouflage of the *E. coli* membrane bolstered the efficiency of phagocytosis while prohibiting the leakage of the drug in neutrophils. Through controlled intravascular movement exposed to a rotating magnetic field, neutrobots can accumulate autonomously in the brain, and then pass through the BBB via positive chemotactic movement along the inflammatory factor gradient. Compared with traditional drug injection, the use of this dual-response neutral robot for targeted drug delivery can arrest tumor cell proliferation greatly [156]. The neutrophils developed in this research afford a promising path for future precision biomedicine. To assist fertilization or to deliver drugs in the reproductive system, Haifeng Xu et al. put forth an integrated system combining a magnetically driven micromotor and a synthetized protein-based hyaluronic acid (HA) microflake for the in situ selection and transport of multiple motile sperm cells. This delivery system can transport not only multiple motile sperms, but also other actively moving biological cargoes [11].

### 3.3. Drug Release Triggered by Different Conditions

Magnetic nanoparticles have become attractive candidates for their nano scale and accumulation in target sites under an external guidance. Jimenez-Lopez C’s group realized targeting and hyperthermia by using biomimetic magnetic nanoparticles (BMNPs) mediated by magnetosomes, with the help of an external gradient magnetic field and alternating magnetic field [157]. However, the difficulty of cell internalization of BMNPs still hinders the efficiency of these nanoparticles. Subsequently, they studied and developed a new technology to produce PLGA–embedding BMNPs. After encapsulated by PLGA, they further functionalized the nanoparticles with the cell-penetrating TAT peptide (TATp), which improved the uptake of BMNPs by the cell [158]. The PLGA-BMNPs in functionalized with TAT peptide enhances the BMNPs cellular uptake without modification of the BMNPs’ magnetic properties and/or their in vitro performance as hyperthermia agents, paving the way for the use of these nanocarriers in combined antitumoral therapy. The application of BMNPs in the magnetic hyperthermia agents is limited. By combination of the BMNPs functionalized with doxorubicin with the irradiation of a laser beam in the near infrared, it can effectively realize the combination of directional therapy and photothermal therapy and increase the toxicity to cells [159]. When functionalized with PLGA and TATp, the nanoparticles are able to mediate both directed chemotherapy and hyperthermia treatment [160]. These works represent the progress of combination therapy in improving the efficiency of antitumor therapy.

## 4. Increasing the Drug Loading Rate

**Figure 4:**
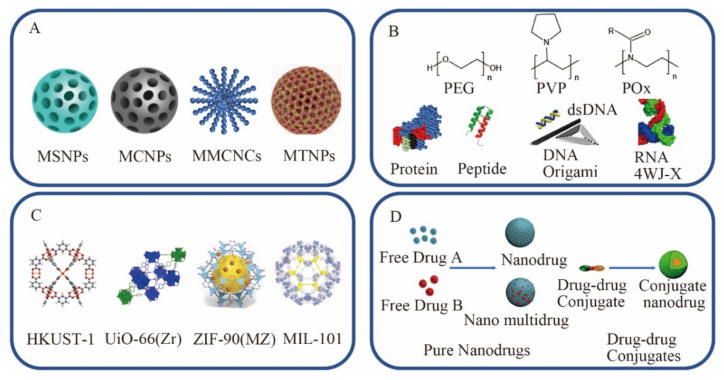
Summary of carrier drugs composed of different carriers. (**A**) Inorganic carrier nano drug delivery system: MSNPs [162,181], MCNPs [160], MCNCs and MTNPs [181]. Reproduced with permission [162,181]. Copyright 2019, Multidisciplinary Digital Publishing Institute and Elsevier. (**B**) Organic carrier nano drug delivery system: PEG, PVP, Pox, protein, peptide [171], DNA [182] and RNA [172]. Reproduced with permission [171,182]. Copyright 2018 and 2012, American Chemical Society. Reproduced with permission [172]. Copyright 2020, Nature Publishing Group. (**C**) MOF carrier nano drug delivery system: HKUST [183], UiO [184], ZIF [185] and MIL [186]. Reproduced with permission [183,184,186]. Copyright 2017, 2014, 2021 and 2018, Elsevier. (**D**) Carrier-free nanomedicines delivery system, including pure nanodrugs and drug–drug conjugates. Reproduced with permission [164]. Copyright 2020, American Chemical Society.

As shown in Table 3, we summarized the advantages and disadvantages of different carrier drugs. According to the different carriers in the delivery system, we expound the drug carrying capacity of different carrier nano drug delivery systems from four parts: inorganic carrier nano drug delivery system, organic carrier nano drug delivery system, MOF carrier nano drug delivery system, and carrier-free nanomedicine delivery system (Figure 4). As summarized in Table 3, we summarize the components, advantages and disadvantages of different carrier drugs. Generally, carrier drugs have the characteristics of low drug loading and potential carrier toxicity, but the presence of carriers enhances the versatility and modifiability of drugs. The carrier-free nanomedicine has the characteristics of high drug loading and carrier-free toxicity, but the problems of low targeting, organic solvent residue and difficulty in surface modification also limit its clinical application.

### 4.1. Inorganic Carrier Nano Drug Delivery System

The improvement of the drug-carrying capacity of inorganic carriers is mainly due to the high surface area and large pore size of porous materials. The current inorganic porous materials include mesoporous silica-based NPs (MSNPs), mesoporous carbon NPs (MCNPs), magnetic colloidal NCs (MCNCs), mesoporous TiO2 NPs (MTNPs), etc. [161]. The advantage of inorganic carrier nano-drug delivery system is increasing the targeting of drugs [162]. In addition, the magnetism, photothermal effects, and ultrasound effects possessed by different inorganic carriers have also contributed a variety of ideas to diagnosis and treatment in the course of disease treatment. However, it is generally believed that NPs may be unstable when passing through different parts of the body, and their high surface energy tends to make them aggregate. Moreover, the protein absorbed on the surface of NPs may not only alter their surface characteristics, but also bring about changes to the protein and possibly their metabolism [163].

#### 4.1.1. Mesoporous Silica-Based NPs (MSNPs)

In 2001, Vallet-Regi et al. introduced a mesoporous silica material called MCM-41, which could be used as a drug carrier. Since then, people have synthesized multifunctional mesoporous silica nanoparticles (MSN) with different nanostructures and morphologies to meet the needs of pharmaceutical and medical applications [187,188]. Mesoporous silica nanoparticles (MSNP) were inherently strong and existent on surfaces with modular symmetry, which could be further modified by chemical functionalization. In addition, the high aspect ratio enhanced surface functionalization, with better porosity to carry molecular cargo without any interference to the silica framework. MSNP’s large Brunauer–Emmet–Teller (BET) surface area (700–1000 m^2^/g) and pore volume (0.6–1 cm^3^/g) also conduced to high drug loading (up to 50% *w*/*w*) so that it could adsorb multiple drug molecules [189]. Peng et al. synthesized a core–shell hybrid nanoparticle composed of a copolymer shell with N-(3,4-dihydroxyphenethyl) methacrylamide (DMA) and N-isopropylacrylamide (NIPAM) as the response part. In the presence of Fe^3+^, a catechol-Fe^3+^ complex was formed to achieve a pH response. Furthermore, poly(N-isopropylacrylamide) also increased the temperature-sensitive specificity of the material. By adjusting the concentration of DOX, the drug loading was easily bumped up from 8.6% to 28.0% [190]. Ozcelik et al. adopted the supercritical carbon dioxide method to load poorly water-soluble carvedilol on spherical silica and MCM-41, with drug loadings of 42% and 26%, respectively [191]. The carrier nanomedicine could protect the drug from degradation and improve its absorption in the intestinal tract. Andreani et al. opted for silica nanoparticles (SiNP) coated with different hydrophilic polymers, namely chitosan, sodium alginate, or low and high molecular weight poly(ethylene glycol) (PEG 6000 and PEG 20000) as a mucosal adhesion carrier for the development of oral insulin. The insulin binding efficiency in SiNP was recorded at more than 70%. After coating, its association efficiency rose to 90%, indicating that the protein had a high affinity for hydrophilic polymer chains. Biofilm model studies have found that, compared with chitosan or sodium alginate, PEG 6000 facilitated higher interactions with liposome polar groups through the formation of hydrogen bonds and/or electrostatic interactions. In addition, PEG 6000 is more effective in protecting insulin from heat denaturation [192]. Silica nanoparticles are also used in the field of oral vaccine delivery. Wu et al. decided on bovine serum albumin (BSA) as a protein antigen model to reveal the characteristics of mesoporous silica nanoparticles coated with chitosan. The encapsulation efficiency and drug loading were 25.34 ± 0.76 and 20.21 ± 0.48%, respectively. The released antigen structure was stable and induced a strong immune response in mice, demonstrating that chitosan mesoporous silica nanoparticles can be utilized as a promising oral vaccine carrier [193]. In addition, silicon-based halloysite is a natural tubular material with a diameter of 50 nm, an inner lumen of 15 nm and a length of 600–900 nm [194]. The drug loading of halloysite nanotubes (HNTs) reaches 10–30 wt. %. Wu et al. designed and synthesized HNTs (HNTs-PEG-FA) combined with poly (ethylene glycol) and folate, and effectively delivered the anticancer drug DOX to breast cancer [195]. DOX is released continuously and controlled for up to 35 h in the acidic environment of tumors, and effectively inhibits the growth of solid tumors.

#### 4.1.2. Mesoporous Carbon NPs (MCNPs)

Mesoporous carbon nanoparticles are widely used to construct nano therapeutic systems, due to their excellent physical and chemical properties. Compared with traditional silica-based nanocarriers, the hydrophobicity of hollow mesoporous carbon nanospheres (HMCNs)–based nanocarriers can achieve higher drug loading efficiency. The high loading capacity should be attributed to the hollow structure of HMCNs, its π–π stacking with the drug and the non-covalent interaction of electrostatic attraction [196]. By introducing near-infrared light irradiation or H_2_O_2_, the movement of HMCNs can be promoted, and the number of HMCNs attached to the surface of cancer cells will grow, which is conducive to improving the efficiency of drug delivery [197]. In the DOX/HMC-Au@PEG system constructed by Zhao et al., the loading capacity of DOX is as high as 40.6%, and the system displays dual-triggered drug release characteristics of redox and NIR [198]. Gui et al. developed a simple and efficient strategy for preparing fluorescent hollow mesoporous carbon spheres (HMCS). Ten drugs commonly used in cancer treatment—including DOX, 5-FU, PTX, CTX, MB, SCC, Ce6, DDP, CUR and QUE—were successfully incorporated into HMCS, with a maximum load efficiency of 42.79 ± 2.7% [199]. Importantly, when combined with 980 nm laser irradiation, it was found that microwaves could improve the photothermal effect produced by HMCS and check the growth of tumor cells. Carbon nanotubes (CNTs) are a carbon allotrope, which have the characteristics of resonance light luminescence, strong NIR optical absorption and Raman scattering, and can be used for cancer multi-modal imaging and treatment [200]. In addition, CNTs can also enhance the anti-tumor effects of chemotherapeutics by improving the accuracy and efficiency of drug delivery [201]. However, the potential long-term toxicity also limits the practical application of CNTs.

#### 4.1.3. Mesoporous Magnetic Colloidal Nanocrystal Clusters (MCNCs)

Mesoporous magnetic colloidal nanoclusters (MCNCs) boast high magnetization, sufficient surface area, excellent colloidal stability, good biocompatibility and acid degradation. Therefore, it is highly anticipated that MCNCs can be used as carriers for targeted drug delivery. Luo et al. described an unprecedented method of synthesizing mesoporous magnetic colloidal nanoclusters (MCNC), which was stabilized by poly (γ-glutamic acid) (PGA), with high magnetization, large surface area (136 m^2^/g) and pore volume (0.57 cm^3^/g). The prepared mesoporous MCNCs are used as hydrophobic drug delivery carriers (paclitaxel as a model drug), with their loading capacity being as high as 35.0 wt% [202]. Sun et al. used the nano-precipitation method to load DOC into MCNCs and achieved high drug loading of 24 wt% [203].

#### 4.1.4. Mesoporous TiO_2_ NPs (MTNPs)

TiO_2_ is widely used in the semiconductor field and biomedical field for its low cost, satisfactory biocompatibility, environmental protection and chemical durability. As a new family member of TiO_2_-based materials, mesoporous TiO_2_ (mTiO_2_), characterized by low cytotoxicity and high mesoporous volume, was constructed as a good drug carrier in biomedical applications. In order to compensate for the shortcomings, such as poor drug carrying capacity, He et al. prepared a multifunctional nanocomposite that integrated mesoporous TiO_2_ nanoparticles (mTiO_2_s) with the promising photothermal material polypyrrole (PPY) to exert a synergistic effect on the treatment of tumors. The results showed that mTiO_2_@PPY-HNK had multi-therapeutic effects and bimodal imaging characteristics, with its drug loading capacity at 6.5%, ±0.3% [204]. However, traditional pure TiO_2_ nano-compounds suffer from problems, such as low drug loading capacity, limited ultraviolet tissue penetration, and 980 nanometer NIR heating effect on normal tissues. In an effort to overcome these problems, Ren et al. designed a new type of mesoporous silica (mSiO_2_)–coated black TiO_2_ core–shell nanocomposite as a pH-responsive/near-infrared accelerated DOX release nanocarrier, thereby improving and broadening the applications of TiO2 nanoparticles in nanomedicine [205]. The results demonstrated that the drug loading efficiency of DOX was 5%, which is 10 times higher than that of bare B-TiO2.

### 4.2. Organic Carrier Nano Drug Delivery System

The organic carrier is composed of synthetic polymers, such as poly(ethylene glycol) (PEG), poly(vinylpyrrolidone) (PVP) and polyoxazoline (POx), as well as natural biopolymer materials, such as proteins, peptides and nucleic acids. Compared with inorganic carriers, its outstanding advantages are that it does not contain inert carrier materials, reduces costs and toxicity risks, and has better biocompatibility.

#### 4.2.1. Synthetic Polymer

PEG has been approved by the U.S. Food and Drug Administration for use in humans. It is the most commonly used hydrophilic component in polymer micelles, ranging from 500 Da to 20,000 Da in molecular weight. The surface modification of PEG-based nanocarriers can reduce the interaction with plasma proteins and the non-specific uptake of the reticuloendothelial system and prolong the circulation time [166]. In a move to tackle the problem of the release of vincristine sulfate (VCS) at the tumor site, Bakmaz et al. prepared and characterized chitosan (CHS)-polyethylene glycol (PEG)-oleic acid (OA) composite materials for the delivery of the VCS carrier and achieved a loading efficiency of 64.1 ± 0.6% [206]. However, the non-biodegradability of high molecular weight PEG and the accelerated blood clearance (ABC) phenomenon caused by repeated injections in the body restrict the use of PEG [207]. PVP has become a good substitute for PEG because of its absence of blood clearance (ABC). The amphiphilic spherical polymer micelle is composed of a core and a hydrophilic shell formed by the aggregation of hydrophobic segments, and thus the hydrophobic drug can be efficiently loaded into the core of the micelle and dissolved in an aqueous medium. Zhai et al. used hydrophobic block poly(ε-caprolactone) (PCL) and hydrophilic block poly(N-vinyl-2-pyrrolidone) (PVP) to synthesize and characterize an amphiphilic biodegradable star-shaped block copolymer TEA(PCL-b-PVP)3. With folic acid as a model drug mixed into TEA (PCL-b-PVP) 3 micelles, the drug loading and encapsulation efficiency were 16.36% and 49.08%, respectively [167]. POx is also called “pseudopeptides” as a result of its structure similar to poly (amino acid) [168]. Similarly, it has become another substitute for PEG, due to its biocompatibility, high degree of solubility adjustment, size change, structure and chemical function. Its five-membered heterocyclic monomers include 2-methyl-2-oxazoline (MeOx), 2-ethyl-2-oxazoline (EtOx), 2-isopropenyl-2-oxazoline (iPrOx) 2-phenyl-2-oxazoline (PheOx) or syntheses from nitriles, carboxylic acids, aldehydes or MeOx [208]. Dong et al. prepared a series of amphiphilic POx block copolymers with various functional groups and studied the relationship between functional structure and drug loading capacity [209]. By encapsulating eight commonly used drugs with various characteristics, including olaparib, paclitaxel, BLZ945, celecoxib, DOX, imiquimod, tranilast, and obeticholic acid, in POx polymers, the author found that, compared with PMBEOx, the drug loading capacities of DOX and imiquimod in PMBEOx-COOH were significantly increased to 18.5% and 10.9%, respectively. In addition, polyelectrolyte microcapsules with a hollow shell structure have also received extensive attention. The polyelectrolyte microcapsules are assembled using the layer-by-layer (LbL) technique, and contain two basic components: the core template and the polyelectrolyte pair [210]. After the core template is dissolved, the drug can be loaded into the hollow polymer shell. Shen et al. designed a biodegradable chitosan–alginate polyelectrolyte multilayer capsule and packed DOX into a BSA gel capsule [211]. Compared with free DOX, BSA-gel capsules loaded with DOX showed better breast cancer treatment effect after treatment.

#### 4.2.2. Natural Biopolymers

In the review by Chen et al., they expressed their concerns about the polydispersity and potential solvent toxicity during the chemical synthesis of synthetic polymers [3]. Most natural polymers are endogenous substances, and because they can be naturally metabolized through physiological pathways, they exhibit better biocompatibility and safety.

As integral biomolecules that make up the body, proteins are essential for maintaining the normal functions of the body. As a drug delivery carrier, protein has garnered much attention, due to its high biocompatibility, non-antigenicity, good biodegradability and easy surface modification [169,170]. Camptothecin (CPT) and curcumin (CCM) are functionalized with 2-acetylphenylboronic acid (2-APBA) and then combined with bovine serum albumin (BSA) through the formation of iminoborate, resulting in high loading efficiency and colloidal stability nanoparticles. At the same time, its pharmacokinetics has also been significantly improved. Nanoparticles effectively release drugs in the specific microenvironment of the tumor [170]. There are approximately 650,000 protein–protein interactions (PPIs) in the human proteome, which do not provide potential therapeutic targets for various diseases. Peptides are ideal candidates for PPI inhibitors because they have a large interaction interface rich in chemical and structural diversity [171]. However, the characteristics of instability in vivo and weak membrane penetration hinder its application. Hong et al. researched and synthesized peptide-modified star-shaped polymers (PET-CL-P) and PEGylated star-shaped polymers (Tri-CL-mPEG) as carrier materials, and selected ACP-GPLGIAGr9-ACP as functional peptides. Using protein endonuclease specific shear peptide (GPLGIAG), a targeting element that can be specifically recognized and sheared by MMP-2 and MMP-9.36, the resulting cationic CPP polyarginine r9 can enhance the penetration of nanoparticles into cells. The drug loading is 5.38 +/− 0.316%, while the cumulative release value in the acidic micro-environment of the tumor is 98.12% [53].

DNA nanoparticles are endowed with the advantages of high structural programmability, high biocompatibility, customizable shapes, and large volume. The emerging DNA nanostructure carriers have been extensively explored in drug delivery and disease treatment [212]. Jiang et al. used DNA origami to achieve DOX loading efficiency of up to 50–60%. The DNA origami carrier facilitates the absorption of DOX in the anti-DOX human breast adenocarcinoma sub-cells (res-MCF 7) and participates in the avoidance of drug resistance [182]. RNA nanoparticles can be constructed via bottom-up self-assembly, with good biocompatibility and precise controllability of composition, structure and function. Guo et al. reported an RNA cross-linked nanoparticle with ultra-thermodynamic stability to dissolve and load paclitaxel for targeted cancer therapy, which addressed the problem of poor water solubility and high toxicity. Here, each RNA nanoparticle can be covalently loaded with 24 paclitaxel molecules as prodrugs, and the formed RNA–paclitaxel complex is structurally rigid and stable. Using RNA nanoparticles as a carrier bolsters the water solubility of paclitaxel by 32,000 times [172]. In the case of achieving a good tumor treatment effect, undetectable toxicity or immune stimulation, although problems still abound in cancer treatment and diagnosis, DNA, RNA and peptide therapy using nanomedicine present a very ideal combination. Zhu et al. reported that self-assembled intertwined DNA-RNA nanocapsules (iDR-NCs) could effectively deliver synergistic DNA CpG and short hairpin RNA (shRNA) adjuvants and tumor-specific peptide neoantigens to lymph nodes. The antigen presenting cells (APC) are used in cancer immunotherapy. The iDR-NC/neoantigen nanovaccine produces 8 times more neoantigen-specific peripheral CD8 (+) T cells than CpG, induces T cell memory, and largely inhibits the progression of neoantigen-specific colorectal tumors [213].

### 4.3. MOF Carrier Nano Drug Delivery System

The metal–organic framework (MOF) is a new type of hybrid material formed by the coordination and coupling of a metal core and an organic bridge segment. The integration of both organic and inorganic components bestows upon it many unique advantages. Large surface area, high porosity, adjustable pore size, easy functionalization and biodegradability make it a promising drug delivery platform with high drug loading [173,174]. The current MOF moldings include metal–organic framework Cu-BTC (HKUST), universitetet i oslo (UiO), zeolitic imidazole framework (ZIF) and materials of institute lavoisier (MIL). Li et al. devised a 5-Fu@ZIF drug delivery system to load the drug molecule fluorouracil, with the drug load reaching 21.1% [214]. The nanoparticles are excellent in lung targeting in that its content in the lung tissues remains above 50% after intravenous injection for one week. The acidic micro-environment at the tumor site causes more than 80% of the drug molecules to be released within 4 h, and the animal survival rate surges from 12.5% to 75%. Ke et al. synthesized a Fe_3_O_4_@MIL-100 (Fe) magnetic nanoparticle carrier that could target and control drug release. The anti-inflammatory drug ibuprofen (IBU) has a loading amount of 0.31g g^−1^, and in vitro drug release kinetics experiments show that the complete release of IBU is after 70 h [215]. Although MOF particles have displayed outstanding application prospects in the fields of gas storage and separation, heterogeneous catalysis, sensing, environmental purification and drug release, such drawbacks as fragility, insolubility, difficulty in molding, and low compatibility with other materials limit the application of MOF materials [175]. As a drug carrier, MOF’s unique frame structure increases the drug load; however, its toxicity and low biodegradability preclude its clinical application [176].

### 4.4. Carrier-Free Nanomedicines Delivery System

An inherent problem of carrier nanomedicine is that its drug-carrying capacity is still low (usually <10 wt%), which hinders the accumulation of effective drugs and the therapeutic effects of drugs. The drug inertness of the nanocarrier and the excessive chemical treatment during the preparation process will bring potential harm to the body [167,168]. In order to avoid these hazards, carrier-free nanomedicines, whose nanomaterial matrix is mainly composed of active pharmaceutical ingredients, were developed. The drug loading of all these types of nanomedicines is usually higher than 80 wt%. As per different building blocks, we divide SDND into pure nano-drugs and drug-drug conjugates for discussion [161,164].

#### 4.4.1. Pure Nanodrugs

As of May 2017, the U.S. Food and Drug Administration (FDA) has received more than 80 applications for drug products containing nanocrystals [177]. Since the nanocrystalline medicine does not need a carrier, it has the benefits of high drug loading, not being restricted by the encapsulation rate, and a wide range of drug dosage adjustments. In addition, the process of drug nanometerization helps to improve the utilization efficiency of poorly water-soluble drugs. At the same time, it can be made into capsules, tablets or injection-type freeze-dried powders and other dosage forms, which are convenient for industrial production. Based on the great potential of the drug, various drug delivery methods, such as oral, intravenous, pulse, ocular and dermal drug delivery were formulated. Among them, nanocrystals of budesonide, baicalein and itraconazole have yielded outstanding and successful results in lung drug transport [178]. Zhang et al. used alumina (AAO) as a template to synthesize pure nanodrugs with reproducibility, homogeneity, high production efficiency, and 200 nm adjustable size. In order to counter the issues of drug damage and Al contamination in the processing of nanodrugs, ice was chosen as a green template. Taking advantage of the features of ice particle boundaries containing relatively mobile water molecules, curcumin with high water solubility is applied to the treatment of lung cancer [59,208].

#### 4.4.2. Drug–Drug Conjugates

Drug–drug conjugates are usually self-assembled from multiple drugs. These drugs can be complete therapeutic ingredients or contain small amounts of non-therapeutic ingredients (synthetic polymers or natural biopolymers). In the previous article, we discussed the organic carrier nano-drug delivery system, so next, we will mainly delve into the drug–drug-combined pure nano-drug containing complete therapeutic ingredients. Yan’s research group synthesized an amphiphilic drug conjugate (ADDC), using the hydrophilic anticancer drug irinotecan (Ir) and the hydrophobic anticancer drug chlorambucil (Cb) through hydrolyzable ester bonds [179]. After cell internalization, the ester bond between the drugs was hydrolyzed, releasing free Ir and Cb. Compared with the free drug, this drug showed a longer blood retention half-life, and at the same time effectively overcame the multidrug resistance (MDR) of tumor cells, exhibiting excellent anti-cancer activity. In another article, the hydrophilic floxuridine (FUDR) and the hydrophobic anti-angiogenic drug pseudolaric acid B (PAB) were conjugated to obtain the ADDC compound FUDR-PAB via one-step esterification and conjugation, which also manifested excellent anti-cancer activity [181]. However, the coupling of hydrophilic and hydrophobic drugs limited the application of ADDC. Furthermore, the changes in the molecular structure of the drug during the synthesis process may have a potential impact on the anti-cancer efficacy and pharmacokinetics [161].

## 5. Conclusions

With the development of nanotechnology, nanomedicine is regarded as a potential way to treat diseases, including tumors. This review focuses on summarizing the latest advances in nanoparticle optimization for drug delivery from the perspective of drug-loading strategies. First of all, the increase in biocompatibility can prevent it from being quickly eliminated by the body’s immune system. Then, nanoparticles can promote their preferential accumulation at sites of interest and increase their effectiveness through improved targeting efficiency. Finally, according to the different carriers in the delivery system, we expound the drug carrying capacity of different carrier nano drug delivery system from four parts: inorganic carrier nano drug delivery system, organic carrier nano drug delivery system, MOF carrier nano drug delivery system, and carrier free nanomedicine delivery system. The presence of the carrier provides an infinite matrix of nanoparticles with different properties, which allows nanomedicine to provide easy surface modification, targeted drug delivery and imaging functions in the physiological system. However, low drug loading, poor pharmacokinetics and potential carrier toxicity limit the application of carrier drugs. Therefore, the FDA takes a rigorous attitude toward the approval of carrier drugs. However, pure nanodrugs have no carrier toxicity because they do not contain a carrier. At the same time, they have the advantage of a high drug loading rate, are not restricted by the encapsulation rate, and have a wide range of drug dosage adjustment. However, problems, such as low targeting, residual organic solvents and difficulty in surface modification also limit their clinical application. In summary, we must objectively look at the advantages and disadvantages of different nanomedicines, optimize the design, and integrate the advantages of different nanomedicines. In addition, we have noticed that the nanoparticles in the environment may have some toxicity. Nanoparticles can be inhaled or ingested, accumulated in the organism, and diffused to various tissues and organs, resulting in toxicological effects, such as oxidative stress, apoptosis and lipid peroxidation [216]. However, we believe that with in-depth research and the gradual improvement of nanomedicine, its application in the clinical field will become more extensive.

## Figures and Tables

**Table 1:** Examples of the applications of liposomes with different ligands.

Ligand		Application	Ref.
Peptide	Muramyl tripeptide (MTP)	Melanoma treatment	[23]
	Arginine-glycine-aspartic acid (RGD)	Brain delivery	[24]
	N-formyl-methionine-leucine-phenylalanine (fMLP)	Leishmaniasis treatment	[25]
	Ac-KGFGGGLK peptide	Atherosclerotic detection	[26]
	CGP 31362	Tumor destruction	[27]
	Muramyl dipeptide	Immunomodulating	[28]
	TD peptide	Melanoma treatment	[29]
	p18-4 (WxEAAYQrFL)	Breast cancer treatment	[30]
Antibody	Integrinβ6 monoclonal antibody	Colon carcinoma treatment	[31]
	anti-HER2 monoclonal antibody	Breast cancer treatment	[32]
	Human epidermal growth factor (hEGF)	Skin therapy	[33]
		Breast cancer treatment	[34]
	Programmed Death Ligand-1 monoclonal antibody (α-PD-L1)	Melanoma treatment	[35]
	fibroblast growth factor (FGF) ligands	Bladder cancer targeting	[36]
	Frizzled 10 (FZD10) antibody	Colorectal cancer treatment	[37]
	CD123/CD33 dual-antibody	Reduction of antigen-negative escape	[38]
	CD123 antibody	Targeting to acute myeloid leukemia cells	[39]
	CD44 antibody	Imaging and therapy of hepatocellular carcinoma	[40]
Others	Natural STAT3 inhibitors	Tumor immunotherapy	[41]
	CRISPR/Cas9	Gene silencing efficiency enhancement	[42]
	Aβ-targeting ligands	Alzheimer treatment	[43]
	STING Agonists	Cancer immunotherapy	[44]
	Itraconazole	Enhanced gene delivery of pDNA and siRNA	[45]
	Deoxyribonucleic acid (DNA)	Gene’s carriers in transfection assays	[46]

**Table 2:** Examples of the applications of nanomaterials coated with cell membranes.

Cell Membrane	Nanoparticle	Application	Ref.
Macrophage	silica nanocapsules	4T1 Subcutaneous tumor treatment	[56]
	Au Nanoshells	4T1 Subcutaneous tumor treatment	[57]
	NaYF4:Yb,Er@NaYF4	4T1 Subcutaneous tumor treatment	[58]
	copper sulfide nanoparticles	An allograft tumor of breast cancer treatment	[59]
	Emtansine liposomes	4T1 metastasis lung cancer treatment	[60]
	ROS-responsive nanoparticles	Cardiovascular Disorders treatment	[61]
	ROS-sensitive β-cyclodextrin	Ulcerative colitis treatment	[62]
	Polymeric cores	Acute pancreatitis treatment	[63]
	mPEG5K-b-PLGA11K@miR199a-3p	Myocardial infarction treatment	[64]
Erythrocyte	Polymeric nanoparticles	Biomimetic delivery platform	[65]
	Fe_3_O_4_ nanoparticles	Reducing reticuloendothelial system uptake	[66]
	Gold nanocages	Photothermal therapy	[67]
	All-in-one hollow nanoworms (A-Fe/AuAg@PDA)	Combating Focal Bacterial Infection	[68]
	Black phosphorus	Photothermal cancer immunotherapy	[69]
	Chitosan, heparin and Au	Thrombus Therapy	[70]
	Zinc phthalocyanine and ICG	Photodynamic/photothermal theranostics	[71]
Platelets	Porous nanoparticles	Targeted antitumor drug delivery	[72]
	Polymeric nanoparticles	Reversing thrombus in mouse models	[73]
	PLGA and Fe_3_O_4_ nanoparticles	Dual targeted thrombolytic therapy	[74]
	γ-Fe_2_O_3_ nanoparticles	Ischemic Stroke treatment	[75]
	Malaria protein VAR2CSA	Targeted treatment of primary and metastatic Cancer	[76]
	Liposomes	Targeted therapy of atherosclerosis	[77]
	Photodynamic nanoparticle	Photodynamic therapy	[78]
Stem cell	Nanogels	Tumor targeted drug delivery	[79]
	Fe_3_O_4_ nanoparticles	Cartilage regeneration	[80]
	β-NaYF4:Yb3+,Er3+	Photodynamic therapy	[81]
	Isotretinoin	Acne treatment	[82]
Cancer cell	Glucose oxidase (GOx) and porphyrin metal-organic framework (MOF)	Cancer targeted starvation and photodynamic therapy	[83]
	Upconversion nanoparticles	Imaging of triple-negative breast cancer	[84]
	Mesoporous silica nanoparticles	Regulating drug release	[85]
	MnO_2_ nanoreactor	Combined photodynamic-starvation therapy	[86]
	Immunostimulatory adjuvant	Eliciting multiantigenic antitumor immunity	[87]

**Table 3:** Summary of advantages and disadvantages of carrier and carrier-free drug delivery.

Strategy	Component	Advantage	Disadvantage	Refs
Inorganic carrier nano drug delivery system	MSNPs; MCNPs; MCNCs; MTNPs	Good drug targeting; potential imaging capabilities	Low drug loading; potential carrier toxicity	[161,162,163,164,165]
Organic carrier nano drug delivery system	Synthetic polymer (PEG, PVP, POx); Natural biopolymers (proteins, peptides, nucleic acids)	Good drug targeting; low carrier toxicity; potential imaging capabilities; good biocompatibility	Low drug loading; carrier cleared quickly; poor stability	[3,161,164,165,166,167,168,169,170,171,172]
MOF carrier nano drug delivery system	HKUST; UiO; ZIF; MIL	Good drug targeting; potential imaging capabilities; good biocompatibility	Low drug loading; potential carrier toxicity	[161,164,165,173,174,175,176]
Carrier-free nanomedicines delivery system	Pure Nanodrugs; Drug–Drug conjugates	High drug loading; no carrier toxicity	Poor drug targeting; residual organic solvent	[161,164,165,177,178,179,180]

## Data Availability

No new data were created or analyzed in this study. Data sharing is not applicable to this article.
